# P-1688. Differences in Guideline-Concordant Antibiotic Prescribing Based on Age, Sex, Race, and Ethnicity for the Management of Inpatients with Community-Acquired Bacterial Pneumonia (CABP) due to *Pseudomonas aeruginosa*: a Retrospective Cohort Analysis of the *All of Us* Database

**DOI:** 10.1093/ofid/ofae631.1854

**Published:** 2025-01-29

**Authors:** Corbyn M Gilmore, Adriana M Vargus, Christopher R Frei

**Affiliations:** University of Texas at Austin & University of Texas Health Science Center at San Antonio, Fair Oaks Ranch, Texas; University of Texas at Austin and UT Health San Antonio, San Antonio, Texas; University of Texas, San Antonio, Texas

## Abstract

**Background:**

This is one of the first studies to evaluate health disparities in antibiotic prescribing for inpatients with community-acquired bacterial pneumonia (CABP) from *Pseudomonas aeruginosa*.

**Table 1.**
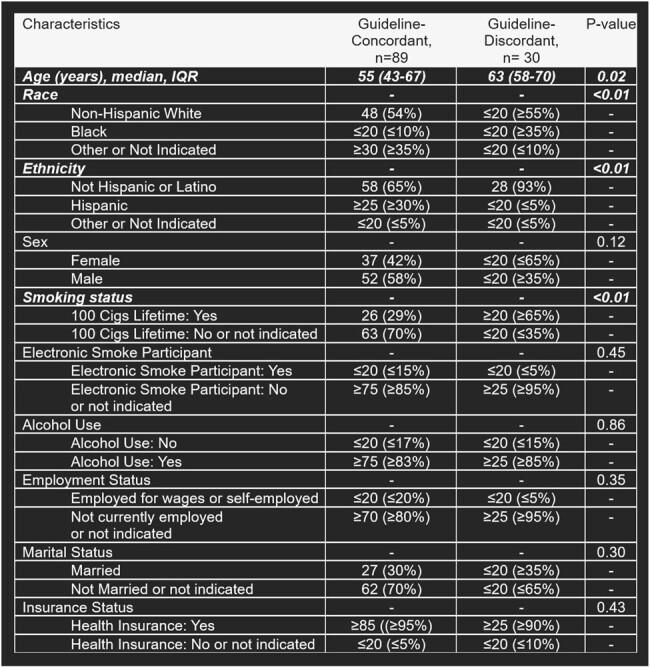
Patient Baseline Characteristics, by Guideline-Concordant and Discordant Antibiotics

* Bold italics indicates statistically significant findings.

+ Some groups contain non-exact participant counts to prevent displaying any participant count that is less than 20 and to comply with the All of Us data and statistic dissemination policy.

**Methods:**

Inpatients with CABP due to *P. aeruginosa* (SNOMED: 41381004) were identified in the *All of Us* database (2016-2022). Patients with treatment settings other than inpatient, other pathogens, and other pneumonia types were excluded. Patients were grouped by age, sex, race, and ethnicity. The proportion of patients prescribed guideline-concordant and guideline-discordant anti-pseudomonal antibiotics (GCAB and GDAB) were compared with chi-square. GCAB (2019 IDSA/ATS CABP guidelines), were: aztreonam, cefepime, ceftazidime, imipenem, meropenem, and piperacillin-tazobactam. Significant associations between patient characteristics and prescribing (p < 0.05) were assessed using multivariate logistic regression, with subgroup as the independent variable, anti-pseudomonal prescribing as the dependent variable, and divergent baseline characteristics as confounders. Odds ratios (OR) and 95% confidence intervals (95% CI) for receipt of GCAB were calculated.

**Table 2.**
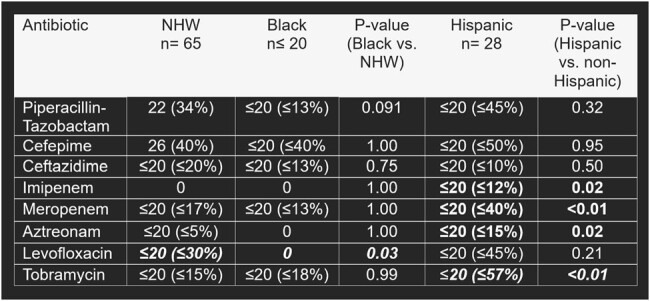
Antipseudomonal Medications, by Race and Ethnicity

* Bold italics indicates statistically significant findings.

+ Some groups contain non-exact participant counts to prevent displaying any participant count that is less than 20 and to comply with the All of Us data and statistic dissemination policy.

**Results:**

A total of 119 patients with CABP from *P. aeruginosa* were included, and 79% received GCAB. Age (p=0.02), race (p < 0.01), ethnicity (p=0.01), and smoking status (p < 0.01) differed between GCAB and GDAB groups. Non-Hispanic White (NHW) patients were more likely to receive GCAB than Black patients (adjusted OR, 6.60; 95% CI 1.75-29.60; p < 0.01). Non-Hispanic (NH) patients were less likely to receive GCAB than Hispanic patients (aOR, 0.12; 0.006-0.65; p = 0.04). Smokers were less likely to receive GCAB than nonsmokers (aOR, 0.20; 0.06-0.61; p < 0.01). Black patients were less likely to receive levofloxacin (aOR, 0.01; < 0.01-0.19) than NHW patients and NH patients were less likely to receive aztreonam (aOR, 0.07; < 0.01-0.52), imipenem (aOR, 0.08; < 0.01-0.72), meropenem (aOR, 0.24; 0.09-0.64), and tobramycin (aOR, 0.13; 0.05-0.35) than Hispanic patients.

**Table 3.**
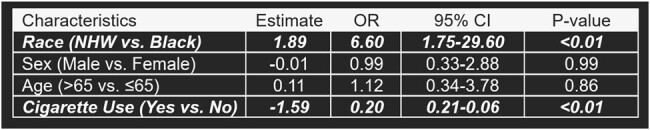
Logistic Regression Analysis of Race and Guideline-Concordant Antipseudomonal Prescribing

* Bold italics indicates statistically significant findings.

** Patients who did not provide an indication for any of the above characteristics were excluded from this model (n=38).

**Conclusion:**

Significant differences in prescribing patterns existed by age, race, ethnicity, and smoking status. Evaluation of the implementation of current guidelines is necessary to ensure timely administration of effective therapy and improve patient outcomes.

**Table 4.**
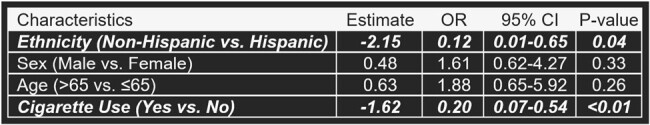
Logistic Regression Analysis of Ethnicity and Guideline-Concordant Antipseudomonal Prescribing

* Bold italics indicates statistically significant findings.

** Patients who did not provide an indication for any of the above characteristics were excluded from this model (n≤20).

**Disclosures:**

**Christopher R. Frei, PharmD, FCCP, BCPS**, AstraZeneca: Grant/Research Support

